# Say you’ll be there: Associations between observed verbal responses, friendship quality, and perceptions of support in young adult friendships

**DOI:** 10.1177/02654075231195115

**Published:** 2023-08-17

**Authors:** Erin P. Macdonald, Thomas H. Khullar, Ella L. Vezina, Katya Santucci, John E. Lydon, Amanda J. Rose, Melanie A. Dirks

**Affiliations:** 1 Department of Psychology, 5620 McGill University, Montreal, QC, Canada; 2 Department of Psychological Sciences, University of Missouri, Columbia, MI, USA

**Keywords:** Friendship, friendship quality, social support, supportive communication, young adult

## Abstract

Friendships are a primary source of social support during young adulthood; however, little is known about the factors associated with young adults feeling greater support during interactions with friends. We examined how micro-level verbal responses and macro-level judgments of friendship quality were associated with perceptions of support following an interaction between friends. Same-gender friend dyads (*N* = 132; 66.2% female; 18–24 years, *M* age = 19.63) took turns speaking about a problem, then participants rated their perceptions of support given and received following the task. We coded each participant’s verbal responses while in the listening role. Actor Partner Interdependence Models (APIMs) revealed significant partner effects for negative engagement responses, such that greater negative engagement responses were linked with the partner perceiving poorer support both given and received. Models revealed significant actor effects for supportive responses, such that greater supportive responses predicted the actor perceiving better support both given and received. Additionally, models revealed significant actor effects of friendship quality predicting actors’ perceiving better support both given and received. Finally, exploratory models revealed minimal interactions between a few types of verbal responses and positive friendship quality. Taken together, results suggest that (a) negative verbal responding styles may be more meaningfully associated with partners’ perceptions of support in the moment than are supportive behaviours, whereas (b) supportive verbal responding styles may be more meaningfully associated with actors’ perceptions of support in the moment, and (c) actors’ judgments of friendship quality are strongly associated with their overall perceptions of support, and a critical factor to consider in future research.

## Introduction

Friendships are an important and understudied relationship in young adulthood (defined here as ages 18 to 25 years), a period during which friendships become a primary source of social support (Carbery and Buhrmester, 1998). While support from friends plays an important role in young adult well-being (Friedlander et al., 2007), young adults also report that providing support is a key challenge occurring within their friendships (Kirmayer et al., 2021), suggesting that effective provision of support may be difficult for some young adults. As such, examining interactions between friends may provide key insights into the support process as it unfolds within young adult friendships.

Research on supportive communication has identified globally effective forms of supportive responding, such as verbal person-centredness, typically utilizing trained confederates as the support providers. However, much less is known about how the broader array of verbal responses that occur during actual interactions between friends are linked to in-the-moment perceptions of support. Moreover, minimal work has sought to understand the contribution of relationship quality to the exchange of support. While relationship quality has been highlighted as a critical factor relating to perceptions of support during interactions between married couples (e.g., Priem et al., 2009), young adult friendships differ from marriages in important ways that may shape the relative importance of relationship quality and supportive behaviours during an interaction to perceptions of support.

The overarching goal of this study was to understand how micro-level verbal responses to a same-gender friend discussing a problem, as well as macro-level perceptions of the positive features of the relationship predict young adults’ perceptions of support following an interaction. Based on prior research, we anticipated that coded verbal responses during the interaction would be linked to the actors’ perceptions of support given and the partners’ perceptions of support received, and that higher levels of positive friendship quality would be associated with better perceptions of support for the actor.

### Social support in young adult friendships

The period of young adulthood from ages 18 to 25 years, commonly referred to as emerging adulthood, is characterized in Western societies by transitions and challenges including attending university or college and moving away from home (Arnett, 2007). During young adulthood, high-quality friendships are a critical relationship linked to increased well-being (e.g., Buote et al., 2007). Social support may underlie the benefits of high-quality friendships for young adults. Perceived support, satisfaction with the availability and quality of support in one’s social network, (Zimet et al., 1988), is strongly linked to well-being (e.g., Friedlander et al., 2007; Haber et al., 2007). During young adulthood, friends become primary sources of social support, replacing family as the main support providers (Carbery and Buhrmester, 1998), and social support from friends is more strongly associated with psychosocial well-being than is support from family (Allen et al., 2000). Same-gender friends are particularly prominent providers of support (Carbery and Buhrmester, 1998), as young adults tend to disclose personal difficulties to and seek support from same-gender friendships (Weisz and Wood, 2005).

The provision of social support is a key task within young adult friendships, and it is also a challenging one. Kirmayer et al. (2021) asked young adults to identify situations with same-gender friends that were important, frequently occurring, and difficult to manage. Many of the situations described involved provision of support, with participants reporting dissatisfaction with both the support they received from friends, and uncertainty about how best to provide support to friends.

Although exchanging support appears to play a critical role in the friendships of young adults, minimal research has focused on understanding the support process specifically within young adult friendships. Social support is a highly interpersonal construct (e.g., Lakey and Orehek, 2011), primarily occurring during interactions within the context of relationships. Thus, examining interactions between friends may provide insight into the specific behaviours and contextual relationship factors associated with perceptions of support.

### Verbal responses and support perceptions

Verbal communication is a primary channel through which support is conveyed. Research on supportive communication has focused extensively on verbal person-centredness, or the overall extent to which a message expresses care, concern, validation, or encouragement, aimed at reducing the distress and improving the emotional state of the support recipient (e.g., Burleson, 2003). These messages have been linked to effective support provision and emotional improvement in the support recipient (High and Dillard, 2012). While verbal person-centredness has been identified as an effective form of verbal responding, much of this work relies on participants disclosing problems to confederates trained to respond with either different levels of verbal person-centredness (e.g., Jones, 2004), or with active listening skills (e.g., Bodie et al., 2015). Thus, while the existing research suggests that this globally supportive responding style is linked to better outcomes by the support recipient, we know less about the specific verbal responses that occur during actual interactions. Recent work by Solomon et al. (2021) noted the consensus that conversational partners comprise an interdependent and mutually influential system, such that each partner is influenced by the thoughts and actions of the other. Outcomes of the interaction are therefore a by-product of the dyadic exchange. Despite the importance of these dyadic factors, there is surprisingly scant research on what happens between partners during interactions to produce those conversational outcomes. As such, research should focus on the broader array of verbal responses that may occur during supportive interactions between friends.

Another important consideration is how supportive communication is measured. To date, research examining levels of person-centeredness has typically measured at a macro level, for example, rating an entire interaction with a confederate as either high, moderate, or low in person-centredness. However, these global ratings may not be an entirely accurate reflection of the nuances that occur within actual interactions. Examples of research examining micro-level coding of dyadic interactions, such that individual utterances or speaking turns are independently coded, are relatively rare. Moreover, such studies have not linked micro behaviors to perceptions of support. For example, research examining supportive interactions between pairs of friends found that ratings of high verbal person-centredness were linked to specific responses such as legitimizing the situation, asking about the friend’s emotional state, and the support provider describing an action they would take in the same situation (MacGeorge et al., 2019). Furthermore, research examining verbal responses during interactions within adolescent friendships have also identified both positive conversational engagement responses, including offering direct support or agreement with the friend, or responses acknowledging the friend has spoken, as well as negative conversational engagement responses, such as criticizing, blaming, changing the subject, or minimizing the problem (Rose et al., 2016).

While these findings provide some insight on the specific messages that may occur within interactions between friends, these findings provide limited insight on the link between specific verbal messages and perceptions of support by the interaction partners. Moreover, minimal research has assessed the associations between verbal responses and support perceptions of both the support provider and the support recipient. Given that dyadic interactions are highly iterative, such that behaviours, responses, and feelings are mutually influential (e.g., Solomon et al., 2021), it is therefore critical to understand these interactions from the perspective of both the provider and the recipient. Thus, while verbal responding is a critical factor relating to perceptions of support, research should examine the specific verbal responses linked to perceptions of support within dyadic interactions. Examining micro-level verbal responses that occur during a supportive interaction between friends will provide key insights into the ways in which friends support one another, both in the role of support provider and support recipient. Furthermore, studying these interactions within existing friendships will allow us to better understand the role of relationship quality in the support process.

### Friendship quality and support perceptions

Studying supportive interactions in the context of on-going relationships provides key insights into the support process, as features of the relationship may be associated with perceived support during an interaction. Research with romantic couples has documented a phenomenon referred to as “sentiment override,” in which self-reported relationship satisfaction is more strongly linked to perceptions of support during an interaction than are specific behaviors (Weiss, 1980). For example, better perceptions of support following an interaction between romantic partners were more strongly linked to relationship quality (Gurung et al., 1997) and relationship satisfaction (Priem et al., 2009) than to observable support. While limited work has examined supportive interactions between friends, Guntzviller et al. (2017) found that during interactions in which one person discussed a problem and their friend gave advice, recipients who rated higher relationship closeness also reported greater conversational satisfaction.

While relationship quality may be a critical factor in shaping support perceptions, this conclusion has been supported primarily within romantic relationships. However, romantic relationships differ from friendships in important ways that may shift the relative importance of global relationship features and in-the-moment behaviors. For example, romantic relationships, particularly marriages, may be longer and comprise more frequent interactions compared to young adult friendships, which may result in a stronger link between relationship quality and perceptions of support. Moreover, young adults typically have multiple friends (Krems et al., 2021), and the relative closeness of these individual friendships may change over time, such that friendships are routinely upgraded and downgraded (Langheit and Poulin, 2022). Given these possible differences, young adults may be more diagnostic in their interactions with friends, paying more attention to the ways in which their friend is providing support. To better understand the relative role of behavior in supportive interactions between friends, research should extend beyond relationship closeness, to study how the perceived quality of the friendship is associated with perceived support during an interaction.

Friendship quality comprises the provisions, or benefits, that the relationship confers (Asher and Weeks, 2018). Friendships involve both negative qualities, such as conflict and betrayal, and positive qualities, including companionship and intimacy, which are relatively independent from each other (see Dryburgh et al., 2022; Langheit and Poulin, 2022). Support is a key positive feature of friendship (e.g., Furman and Buhrmester, 1985; Simpkins and Parke, 2001), accordingly, we anticipate that in friendships with more positive features, individuals may feel more supported during interactions with their friends. Accounting for friendship quality may provide a better understanding of the association between observable behaviours and perceptions of support, by allowing us to examine the extent to which verbal responses are uniquely associated with perceived support, after accounting for positive friendship quality.

There are two ways in which positive friendship quality may be associated with perceived support during an interaction. First, theoretically, perceptions of friendship quality are developed through repeated interactions (Hinde, 1995). Presumably, one reason that a friendship is viewed to be high on positive features is because those friends frequently provide each other with support and thus friendship quality reflects positive supportive experiences. As a result, friends’ responses during a supportive interaction should be intimately tied to global perceptions of the positive qualities of the relationship. Second, the quality of the relationship may shape how verbal responses are perceived. For example, prior research asked participants to provide examples of recent interactions with any conversational partner, demonstrating that self-reported closeness in the relationship was linked to greater receptivity to advice from others (Feng and MacGeorge, 2006), and more positive appraisals of a recent hurtful message received from a conversational partner (Young, 2004). These results indicate that positive perceptions of the relationship may have lessened the negative perception of two potentially harmful forms of verbal responding. Accordingly, friendship quality may moderate the association between observable support and perceptions of support during an interaction; in friendships with more positive features observable support actions might be overlooked, whereas in friendships with fewer positive features, observable support actions may be more salient, and thus more strongly associated with perceived support.

### Goals of current study

The overarching goal of this study was to identify how both in-the-moment verbal responses and friendship quality were associated with overall perceptions of support following an interaction between pairs of young adult friends. We observed same-gender friends discussing personal problems, had raters reliably code verbal responses of the listening friend, and asked friend pairs to rate the support they both gave and received immediately following the entire interaction. Using Actor-Partner Interdependence Models (APIMs), we analyzed both actor (i.e., the association between participants’ predictors and their own outcomes) and partner (i.e., the association between participants’ predictors and their friends’ outcomes) effects (Kenny et al., 2006). We addressed the following research questions: (1) What are the associations between verbal responses from the listening friend and (a) the partners’ overall perceptions of support received, and (b) the actors’ overall perceptions of support given, following the entire interaction? (2) How is positive friendship quality linked to the actors’ overall perceptions of support received and support given following the entire interaction? (3) Does the association between verbal responses and actor or partner perceptions of support change as a function of the actors’ report of positive friendship quality?

We hypothesized that we would find partner effects for verbal responses on support received, such that actors’ use of positive verbal responses such as support and acknowledgement would be linked to better support received by the partner, while negative engagement responses would be linked to poorer support received by the partner. We hypothesized actor effects of verbal responses on support given, such that the use of supportive verbal responding would be linked to actors perceiving that they gave better support. Furthermore, we hypothesized actor effects of friendship quality, such that participants reporting greater positive friendship features would perceive better support both received and given. Finally, we anticipated the associations between verbal responses and perceptions of support may be moderated by the actor effect of positive friendship quality, such that verbal responses would be more strongly associated with perceptions of support in friendships in which the actor had reported fewer positive features.

## Methods

### Participants

Participants were 137 same-gender friend dyads (*n* = 91 dyads identified as cisgender women, all other participants identified as cisgender men) between 18 and 24 years (*M* = 19.6, *SD* = 1.34). Demographic information about the sample can be found in Table 1. Participants were recruited through flyers, advertisements posted to Facebook groups, and the Department of Psychology participant pool. All participants were undergraduate students (97.4% full-time) at an English-language university in Montreal, Canada, and 63.9% of the participants reported that their first language was English. On average, dyads had known each other for 32 months (range = one month – 19 years, SD = 41.61 months). Participants received either two extra credits towards a psychology course or were compensated with $20. We excluded data from four dyads due to issues with the video recordings and one dyad due to missing data on the outcome variables, thus, the final analytic sample was *N* = 132 dyads (66.2% female, *M* age = 19.63).

**Table 1.** Demographic information of study sample.

Variable	Total sample (*N* = 274)
Age, *M (SD)*	19.61 (1.34)
Gender identity, *n (%)*	
Female	182 (66.4)
Male	92 (33.6)
Ethnicity, *n (%)*	
White	135 (49.3)
Chinese	33 (12.0)
South Asian	27 (9.9)
African	8 (2.9)
Arab/West Asian	17 (6.2)
Filipino	8 (2.9)
Southeast Asian	7 (2.6)
Hispanic	9 (3.3)
Other^a^	30 (10.9)
Relationship status, *n (%)*	
Single	211 (77.0)
In a relationship	62 (22.6)
Married	1 (.4)
Sexual Orientation, *n (%)*	
Heterosexual	223 (81.4)
Gay or Lesbian	11 (4.0)
Bisexual	35 (12.8)
Other/Prefer not to answer	5 (1.8)
First year of university, *n (%)*	135 (49.3)
Living situation, *n (%)*	
Apartment/house away from family	113 (41.2)
Live in a dormitory	91 (33.2)
Live with parent(s)	61 (22.3)
Live with other family members	7 (2.6)
Live only with romantic partner	2 (.7)

^a^Other category includes participants identifying as Caribbean, Japanese, Korean, participants who selected ‘Other’, and participants who selected ‘Prefer not to answer’.

### Procedure

All procedures were approved by the Research Ethics Board of McGill University. Written informed consent was obtained from all participants. Participants attended a two-hour lab session with a same-gender friend, first completing individual questionnaires assessing interpersonal competence, friendship quality, and other constructs as a part of a larger study. Participants were then audio and video recorded completing the Problem Talk Task (Rose et al., 2016). Prior to this task, participants individually wrote down a current problem they had outside of their friendship and were randomly assigned to a speaking order. During the Problem Talk Task, Participant 1 was instructed to discuss their problem for an eight-minute turn while their friend listened, then participants were alerted by the researcher to switch roles and Participant 2 would discuss their problem for the remaining eight minutes. Following the completion of the entire Problem Talk Task, participants completed individual questionnaires assessing their perceptions of support during the interaction.

### Measures

#### Friendship quality

We used a revised 27-item version of the Friendship Quality Questionnaire (FQQ; Simpkins & Parke, 2001). The FQQ includes items rated on a 5-point Likert scale from 1 (*Not at all true*) to 5 (*Very true*). This scale has 21 items measuring positive friendship quality, such as “My friend cares about my feelings”, and “My friend and I always make up easily if we have an argument”, and six items measuring negative friendship quality. Given that positive and negative friendship quality represent separate constructs rather than opposite ends of a continuum, and our hypothesis that positive friendship quality is more likely to relate to support perceptions, only the positive friendship quality scale was used in the analysis. Participant responses to positive quality items were averaged, with a score closer to five indicating higher positive friendship quality (α = .91).

#### Perceptions of support given and received

Immediately following the completion of the Problem Talk Task, participants answered questions about their perceptions of support during the entire interaction. We created an index of the support that participants *received* from their friend by averaging the following five items rated on a 7-point Likert Scale from 1 (*Not at all*) to 7 (*Extremely*): “I felt supported by my friend”, “I felt better after talking to my friend about my problem,” “I could tell that my friend really cared about me,” “I feel happy about the interaction I just had with my friend,” and “I feel satisfied with our friendship.” Participants’ perceptions of the support they believe their friend felt, referred to here as support *given*, was measured by averaging the same five items re-worded to capture how supported the participant thought their friend felt (e.g., “My friend felt supported by me.”). Alphas were excellent: support received, .83, support given, .84.

### Verbal response coding

Data were coded following a scheme adapted from Rose et al. (2016). To begin, we created verbatim transcripts of the video recordings, removing any identifiable information. Next, each transcript was divided into “thought units”, the unit of analysis for coding. Thought units are a single thought or idea by the same participant, defined by speaker’s intonation, pauses, interruptions, or shifting thoughts, rather than grammar or sentence structure (Rose et al., 2016). Based on independently double coding 25% of the transcripts, there was 90.6% agreement between coders on the start and end point of the thought units. Next, we identified own-problem statements, defined as thought units related to the problem the participant was discussing. Based on independently double coding 25% of the transcripts there was high reliability applying the problem statement codes, *k* = .96.

#### Response coding

Finally, we coded all responses to own-problem statements. First, we highlighted every speaking turn that included an own-problem statement code, and identified the *response* as all thought units within the following speaking turn, indicating that a participant was *responding* to their friend’s problem talk. Each response thought unit was coded into one of seven categories (see Table 2 for code descriptions) based on the most specific code that applied. Only response thought units were coded into these categories. Based on independently double coding 25% of the transcripts there was high reliability applying the response codes, all kappas exceeded *k* = .85 (see Table 2).

**Table 2.** Verbal response codes, descriptions, and coding reliability.

Response code	Code description	Cohens kappa (κ)
Acknowledgment + Prompting	Acknowledging that the friend has been heard, explicitly encouraging friend to say more about their problem	.94
Information + Opinion	Giving an opinion or comment on the problem topic, adding information about the problem topic, or responding with personal experience with the problem topic while maintaining the focus on their friend	.88
Advice + Help	Giving direct advice, either telling their friend what they should do or what they themselves would do, or offering to do some future action to help their friend with the problem	.91
Questions	Asking the friend for more information about the problem topic, asking the friend to repeat themselves, or encouraging the friend to continue speaking about the problem topic	.96
Support	Explicitly conveying support or agreement (positive tone)	.86
Distracting	Response discussing their own experience with friends’ problem topic that draws the attention to themselves	.90
Negative engagement	Explicitly conveying non-support or disagreement, or that the problem is not that important or difficult, changing subject away from the general problem topic, no response from the listening friend, or a substantial break between speaking turns	.85

*Note.* Table based on coding manual developed by Rose et al. (2016).

### Preparing coded data

Several steps were taken to prepare the coded data. The analysis aimed to test associations between responses from the *listening* participants and perceptions of support. During the Problem Talk Task, each participant was in the listening role one time: Participant 2 during the first half of the interaction, and Participant 1 in the second half. Accordingly, only the responses from the participant in the listening role were coded and used in the analysis. In some cases, participants would speak about their own problem while in the listening role, and these thought units had previously been coded as “own-problem statements.” These own-problem statements spoken by the participant in the listening role were recoded as “Distracting” responses, as they indicate the listening participant was speaking about their own problem. Finally, we created proportion scores for each response category for analysis, to account for the total number of thought units in each transcript. Proportions were calculated using the number of responses of a particular type produced by the listening participant, divided by the total number of thought units produced by the participant while in the listening role. Descriptive statistics for the response code proportion scores are reported in Table 3.

**Table 3.** Descriptive statistics of study variables.

Variable	*M*	*SD*	Min	Max
Support received	5.80	.98	1.20	7.00
Support given	5.15	.94	1.20	7.00
Positive friendship quality	4.08	.59	1.95	5.00
Support responses	.09	.08	.00	.51
Acknowledgement + Prompting	.18	.12	.00	.70
Information + Opinion	.27	.14	.01	.74
Advice + Help	.06	.09	.00	.48
Questions	.12	.09	.00	.52
Distracting responses	.07	.12	.00	.78
Negative engagement responses	.02	.03	.00	.26
Total thought units	93.90	33.16	18.00	275.00

*Note.* Verbal response scores presented are calculated as a proportion of the total number of thought units uttered by the listening participant.

### Planned analysis

We used Actor-Partner Interdependence Models (APIMs; Kenny, 2015) to analyse data from 264 participants nested within 132 dyads to test both actor and partner effects. Models specified indistinguishable dyads, given the same-gender friendships within dyads and random assignment of participant speaking order during the task (see Kenny and Ledermann, 2010). Analysis of mean differences between first and second speaker on all study variables revealed one significant finding, however this effect did not retain significance after multiple test correction (see Supplemental Materials Table S1). All independent and dependent variables were mixed; that is, positive friendship quality, verbal responses, and perceptions of support were different between each member of the dyad.

Four APIM models tested the first two research questions. First, two APIM models tested the association between verbal responses and (1) perceptions of support received and (2) perceptions of support given. Then, we added positive friendship quality as a predictor to each model. All APIM models included the seven verbal responses (see Table 2). Covariates included gender (coded 0 = Male, 1 = Female), and whether the participants’ first language was English (coded 0 = Language other than English, 1 = English), which has been associated with perceptions of support received during the Problem Talk Task (Zikic et al., 2022). Finally, we explored whether the association between verbal responses and perceptions of support varied as a function of positive friendship quality, constructing seven models for each dependent variable, including the interaction between positive friendship quality and verbal response type along with the main effects and covariates. All continuous variables were grand mean centred prior to analysis.

## Results

### Analysis of support received and support given

#### Verbal responses

Means and standard deviations for all study variables are reported in Table 3. Pairwise correlations among all study variables are reported in Table 4. The first APIM model tested the association between verbal responses and participants’ overall perceptions of support *received*. Full model results are reported in Table 5. The model revealed two partner effects: distracting responses, *B* = −1.44, *t* (247) = −2.48, *p* = .013; and negative engagement responses, *B* = −4.89, *t* (247) = −2.35, *p* = .019. Both greater distracting and negative engagement responses by the actor were associated with the partner perceiving less support received. Supportive responses showed a significant actor effect *B* = 1.61, *t* (247) = 2.05, *p* = .041, indicating that actors’ using more supportive responses also perceived better support received. Both covariates were significant. Participants reporting English as a first language reported lower levels of support received, *B* = −.28, *t* (247) = −2.16, *p* = .031, and female dyads reported higher levels of support received, *B* = .31, *t* (247) = −2.24, *p* = .026.

**Table 4.** Pairwise correlations of all study variables.

	1	2	3	4	5	6	7	8	9	10	11	12	13
1. Support received	.23**	.70**	.42**	.15*	−.12*	−.02	.02	−.10	.05	.02	.15*	.00	−.10
2. Support given	.21**	.24**	.36**	.15*	−.10	−.01	.05	−.19**	.11	−.05	.10	−.02	−.09
3. Friendship quality	.26**	.17**	.51**	.01	−.14*	.15*	−.01	−.10	−.07	.06	.23**	−.03	.07
4. Support responses	.16*	.12	.03	.30**	.13*	−.06	−.09	−.06	−.17**	−.11	.03	−.01	−.01
5. Acknowledgement + Prompting	−.06	−.06	−.11	.00	.31**	−.18**	−.18**	.30**	−.26**	−.21**	.05	.12	−.01
6. Information + Opinion	.10	−.07	.13*	−.06	−.09	.14*	.09	−.13*	−.24**	−.07	.08	.06	.08
7. Advice + Help	.04	−.00	−.02	.04	−.01	.07	.29**	−.05	−.18**	.07	.09	.09	−.10
8. Questions	−.02	−.07	−.10	−.01	.07	.06	.12	**−.02**	−.29**	−.02	.02	.19**	−.12
9. Distracting	−.16*	.04	−.11	.03	.02	−.08	−.12*	.02	**.07**	−.04	−.08	−.07	−.07
10. Negative engagement	−.13*	−.17**	−.08	−.03	−.06	−.00	−.08	.02	−.20	**.11**	−.11	−.04	.03
11. Gender	.15*	.10	.23**	.03	.05	.08	.09	.02	−.08	−.11	**1**	.03	.09
12. Age	−.00	−.02	−.01	.02	.09	.04	.09	.12	−.01	−.06	.03	.77**	−.15*
13. English first language	−.00	−.01	.15*	−.03	−.04	.03	−.14*	−.04	−.01	−.01	.09	−.14*	.44**

*Note.* Within-dyad correlations for each variable are presented bolded on the diagonal, actor correlations (actor-actor) are reported above the diagonal, and partner correlations (actor-partner) are reported below the diagonal. Gender coded 0 = male, 1 = female. First language coded 0 = language other than English, 1 = English. *p < .05, **p < .01.

**Table 5.** Results of actor-partner interdependence models examining verbal responses predicting support received and given.

	Support received				Support given			
	*B*	*SE*	*t*	*p*	*B*	*SE*	*t*	*p*
Intercept	5.76	.13	43.46	<.001	5.15	.13	40.55	<.001
Support responses *(actor)*	1.61	.79	2.05	.041	1.57	.76	2.05	.041
Support responses *(partner)*	.97	.79	1.24	.216	.49	.76	.64	.523
Acknowledgement/Prompting *(actor)*	−.74	.56	−1.33	.185	−.36	.54	−.66	.511
Acknowledgement/Prompting *(partner)*	−.90	.56	−1.60	.110	−.71	.54	−1.31	.193
Information/Opinion *(actor)*	−.31	.46	−.69	.493	−.00	.44	−.01	.995
Information/Opinion *(partner)*	.22	.45	.49	.625	−.65	.44	−1.49	.139
Advice/Help *(actor)*	−.56	.75	−.75	.454	.46	.73	.64	.523
Advice/Help *(partner)*	−.11	.75	−.15	.881	−.23	.73	−.32	.748
Questions *(actor)*	−.81	.73	−1.12	.265	−1.49	.70	−2.12	.035
Questions *(partner)*	−.33	.72	−.46	.643	−.78	.70	−1.11	.268
Distracting *(actor)*	.16	.58	.27	.789	.55	.57	.97	.331
Distracting *(partner)*	−1.44	.58	−2.48	.014	−.24	.56	−.43	.667
Negative engagement *(actor)*	1.43	2.08	−2.35	.493	−.53	2.01	−.26	.793
Negative engagement *(partner)*	−4.89	2.08	2.24	.019	−5.63	2.01	−2.80	.006
Gender	.31	.14	2.24	.026	.20	.13	1.50	.135
First language	−.28	.13	−2.16	.031	−.22	.12	−1.82	.070

*Note.* Gender coded 0 = male, 1 = female. First language coded 0 = language other than English, 1 = English.

The second APIM model tested the association between verbal responses and participants overall perceptions of support *given* (see Table 5). Negative engagement responses showed a significant partner effect *B* = −5.63, *t* (247) = −2.80, *p* = .006. Actors’ using more negative engagement responses was associated with their friend reporting less support given. The model also revealed two actor effects. Actors who engaged in more supportive responding perceived that they gave their friend better support, *B* = 1.57, *t* (247) = 2.05, *p* = .041. Conversely, actors who asked more questions perceived that they gave poorer support, *B* = −1.49, *t* (247) = −2.12, *p* = .035. Neither first language nor gender were significant covariates in the model.

#### Friendship quality and verbal responses

The third and fourth APIM models tested the association between positive friendship quality and verbal responses on perception of support received and given (see Table 6). Note that we also conducted exploratory APIM models testing the associations between each verbal response individually, positive friendship quality, and support received and given. These results are in Supplementary Materials (Tables S12-S18). In the model examining support *received*, positive friendship quality showed a significant actor effect, *B* = .58, *t* (245) = 5.52, *p* < .001, indicating that actors’ reporting more positive friendship features reported better support received. Negative engagement responses continued to show a significant partner effect, *B* = −3.87, *t* (245) = −1.98, *p* = .048; actors’ using more negative engagement responses predicted their partners reporting less support received. Supportive responses showed a significant actor effect, *B* = 1.61, *t* (245) = 2.18, *p* = .030, indicating that actors’ engaging in more supportive responses perceived better support received. English as a first language was a significant covariate, *B* = −.28, *t* (245) = −2.36, *p* = .019, gender was not a significant covariate.

**Table 6.** Results of actor-partner interdependence models examining verbal responses and friendship quality predicting support received and given.

	Support received				Support given			
	*B*	*SE*	*t*	*p*	*B*	*SE*	*t*	*p*
Intercept	5.90	.12	48.23	<.001	5.26	.12	43.78	<.001
Support responses *(actor)*	1.61	.74	2.18	.030	1.57	.72	2.17	.031
Support responses *(partner)*	.96	.74	1.30	.194	.48	.72	.66	.508
Acknowledgement/Prompting *(actor)*	−.40	.53	−.75	.453	−.07	.52	−.13	.896
Acknowledgement/Prompting *(partner)*	−.55	.53	−1.04	.298	−.42	.52	−.81	.421
Information/Opinion *(actor)*	−.50	.42	−1.17	.241	−.17	.42	−.42	.677
Information/Opinion *(partner)*	.13	.42	.30	.762	−.72	.41	−1.74	.083
Advice/Help *(actor)*	−.21	.70	−.29	.771	.78	.69	1.13	.261
Advice/Help *(partner)*	.19	.70	.28	.783	.02	.69	.03	.972
Questions *(actor)*	−.43	.68	−.63	.528	−1.18	.66	−1.77	.078
Questions *(partner)*	.15	.67	.23	.820	−.35	.66	−.53	.597
Distracting *(actor)*	.52	.55	.94	.346	.84	.54	1.57	.119
Distracting *(partner)*	−.96	.54	−1.76	.080	.18	.53	.35	.730
Negative engagement *(actor)*	.85	1.95	.43	.664	−1.26	1.92	−.66	.512
Negative engagement *(partner)*	−3.87	1.95	−1.98	.048	−4.54	1.91	−2.37	.018
Friendship quality *(actor)*	.58	.11	5.52	<.001	.56	.10	5.38	<.001
Friendship quality *(partner)*	.12	.11	1.15	.250	.04	.10	.34	.733
Gender	.12	.13	.91	.364	.03	.13	.27	.786
First language	−.28	.12	−2.36	.019	−.22	.12	−1.87	.062

*Note.* Gender coded 0 = male, 1 = female. First language coded 0 = language other than English, 1 = English.

In the model examining support *given*, positive friendship quality showed a significant actor effect *B* = .56, *t* (245) = 5.38, *p* < .001, indicating actors’ reporting more positive friendship features perceived giving better support. Negative engagement responses continued to show a significant partner effect *B* = −4.54, *t* (245) = −2.37, *p* = .018, and support responses continued to show a significant actor effect *B* = 1.57, *t* (245) = 2.17, *p* = .031. Neither first language nor gender were significant covariates in the model.

### Interactions between verbal response and friendship quality on perceptions of support

Finally, exploratory models tested whether the association between verbal responses and perceptions of support varied as a function of positive friendship quality. Interaction models utilized a linear mixed model within an APIM framework using the GAMLj package in Jamovi (Gallucci, 2019). No interaction models retained significance after applying the Benjamini-Hochberg false discovery rate correction; however, because these models were exploratory, below we describe the models where the interaction was significant at the conventional alpha of .05. Full model specifications and results can be found in Supplementary Materials.

For models predicting support received, only the interaction between the actor’s negative engagement responses and friendship quality was significant; *B* = 8.39, *SE* = 3.44, *t* (251) = 2.44, *p* = .015 (see Supplemental Table S2). Simple slopes analysis revealed no significance; with all *p* values greater than *p* = .077 (see Supplemental Table S3). Of the models predicting support given, three models returned significant results. Information/opinion responses interacted with positive friendship quality on the partners’ report of support given, *B* = −1.20, *SE* = .56, *t* (255) = −2.13, *p* = .034 (see Supplemental Table S4). Simple slopes were significant both at the mean, *B* = −.81, *SE* = .38, *t* (252) = −2.13, *p* = .034, and at 1SD above the mean of friendship quality, *B* = −1.53, *SE* = .52, *t* (255) = −2.95, *p* = .003 (see Supplemental Table S5). Distracting responses interacted with positive friendship quality on the actors’ perception of support given, *B* = −1.86, *SE* = .80, *t* (253) = −2.34, *p* = .020 (see Supplemental Table S6). Simple slopes were significant both at 1SD below the mean of friendship quality, *B* = 2.11, *SE* = .65, *t* (255) = 3.25, *p* = .001, and at the mean *B* = .99, *SE* = .44, *t* (249) = 2.25, *p* = .025 (see Supplemental Table S7). Negative engagement responses interacted with positive friendship quality on the actor’s perception of support given, *B* = 7.43, *SE* = 3.40, *t* (253) = 2.18, *p* = .030 (see Supplemental Table S2). Simple slopes analysis were significant only at 1SD below the mean of friendship quality, *B* = −7.71, *SE* = 3.38, *t* (254) = −2.28, *p* = .023 (see Supplemental Table S3).

## Discussion

Friendships are a primary source of social support during young adulthood, yet we know relatively little about the ways in which young adults feel supported during conversations with their friends. This study focused on examining how both verbal responses and positive friendship quality were associated with overall perceptions of support following an interaction between friends. Understanding factors associated with feeling supported following an interaction with a friend has numerous implications. Feeling satisfied with the quality of support provided by a friend within a given interaction may contribute to better global perceptions of support quality and availability, which are strongly linked to positive outcomes (e.g., Friedlander et al., 2007; Haber et al., 2007). Furthermore, the ability to provide effective support to a friend, such that the recipient feels more supported, is likely a critical skill for maintaining high-quality friendships. Thus, understanding how support perceptions are shaped within an interaction with a friend might provide insight into improving both the well-being and relationship skills of young adults.

### Verbal responses and support perceptions

We first tested associations between an array of verbal responses to a friend discussing a problem, and perceptions of support both given and received. Consistent with our hypothesis, participants' use of negative engagement responses, such as minimizing the problem, changing the subject, or making directly non-supportive statements (e.g., “That’s dumb”), were associated with lower perceptions of support received by the partner. Similarly, participants’ use of distracting responses were associated with the friend reporting lower perceptions of support received following the interaction, although this finding was not significant when positive friendship quality was included in the model. These findings build on prior work identifying negative styles of verbal responding during conversations between pairs of adolescent friends (Rose et al., 2016). We also found that greater negative engagement by the actor was associated with partners’ perceiving that they gave poorer support, a pattern that suggests that when a friend engages in explicitly negative behavior, the partner may perceive the entire interaction more negatively.

As hypothesized, supportive responses, such as direct agreement (“That’s so true”) or reflecting a friend’s statement back to them, were associated with the actor perceiving better support given following the interaction. In other words, beliefs about giving support were in agreement with observed supportive verbal responses given. This finding builds on the verbal person-centredness literature (e.g., Bodie et al., 2015; Jones, 2004) which has primarily focused on more positive outcomes of verbal person-centredness for the support recipient. Our findings suggest that individually supportive messages may also be aligned with the support providers perceiving a better conversational outcome, in reporting that their friend feels more supported.

Supportive responses were also associated with the actor’s perceptions of better support received following the interaction. This finding may align with the idea that conversational partners mutually influence one another within a dyadic interaction (e.g., Solomon et al., 2021), such that more positive responses from the friend may also lead to more engagement with positive styles of responding, and that participants may engage in more supportive verbal behaviours when they themselves feel more supported. In general, it appears as though actors who feel more support in the interaction, both given and received, also engage in more supportive behaviors.

Supportive responses were not significantly related to partners perceptions of support received, although a review of the zero-order correlations reveals similar actor (r = .15) and partner (r = .16) correlations between supportive responding and support received. It is possible that since models contained all seven verbal response categories, the partner effect of supportive responding may not have emerged in the context of the larger partner effects of negative responses. For the partner, positive verbal responses may not be as salient as negative ones. Given the nature of the interaction task, where participants were asked to each discuss a problem, participants may have expected their friend to respond in an overall supportive manner. Accordingly, positive responses may have met, but not exceeded, this expectation of support. In this case, it may be less likely for neutral or positive behaviours to predict perceptions of support for the partner.

#### Friendship quality, verbal responses, and support perceptions

We also examined the association between verbal responses and perceptions of support both received and given following the interaction, while accounting for participants’ evaluations of positive friendship quality. Consistent with our hypotheses, actors reporting more positive friendship features also perceived better support both given and received following the interaction. This finding aligns with the concept of sentiment override of relationship satisfaction predicting perceived support in romantic couples (Priem et al., 2009; Weiss, 1980), suggesting that positive friendship quality is similarly linked to perceptions of support, even when accounting for verbal responses. Potentially, friendships with more positive features may have a history of good support provision, thus, individuals are more likely to perceive their friends’ responses during the interaction as supportive. Relatedly, young adults reporting more positive friendships may also have a more positive disposition while interacting with their friend, potentially impacting both their style of verbal responding, as well as their overall perceptions of support within the interaction. While evaluations of positive friendship quality appear to be an important factor in the support process, we know little about the way these evaluations are developed within friendships. As such, future research should aim to understand how friendship evaluations are developed within young adult friendships.

Interestingly, although both supportive responses and negative engagement responses retained statistical significance when accounting for friendship quality, both distracting responses and questions were not significantly associated with support perceptions when friendship quality was in the model. These findings highlight the importance of accounting for contextual relationship factors to create a better overall understanding of the support process. It is possible that prior findings regarding the effective communication of support have drawn conclusions about behaviours that may be more accurately attributed to relationship quality. The current findings suggest that accounting for both the behaviours within the interaction, and the quality of the relationship with the person with whom the interaction is occurring, may allow for a more complete picture of the support process.

#### Interaction between friendship quality and verbal responses

Finally, we explored whether the associations between verbal responses and perceptions of support may be moderated by positive friendship quality. None of the interactions between a given verbal response and positive friendship quality retained significance after correcting for multiple testing; however, we did find marginal interactions between positive friendship quality and information responses, distracting responses, and negative engagement responses. Overall, two of the three models suggest that in friendships with fewer positive features, participants’ verbal responses appeared to have a stronger association with their own perceptions of the support they gave during the task. Theoretically, the quality of a friendship may shape the associations between in-the-moment behaviors and perceptions of support; for example, in friendships of lower quality, the presence of positive behaviors may be more salient. As such, it will be important for future research to continue to examine friendship quality as a moderator.

In continuing this line of work, it will be important to recruit friendship dyads of varying quality. Our ability to test friendship quality as a moderator was likely limited by the overall high quality of the friendships within our sample. Given that this research required friends to attend a 2-hour lab session together, we expected a relatively high level of friendship quality within this sample, and indeed, the average positive friendship quality score was close to the maximum score of five (*M* = 4.08). Theoretically, given that social support is a key expectation within high quality friendships, this may have created an overall positive bias in the types of support recorded during the interaction task. In newer or less positively evaluated friendships, we may have seen both greater variation in the style of verbal responding, and greater potential for an interaction between verbal responses and positive friendship quality. Recruiting a sample that comprises friendships of more varied quality may provide greater insight into the extent to which friendship quality may moderate the association between verbal responses and perceptions of support.

#### Limitations and future directions

Taken together, these findings provide insight into the factors associated with perceptions of support within young adult friendships. However, limitations must be noted. Regarding the demographic information of our sample, we did not ask about disability or class information such as family income or socioeconomic status. Furthermore, recruiting same-gender friendships for this research may have unintentionally excluded non-binary or gender nonconforming individuals from our study, which limits the generalizability of our results.

While a strength of this study was measuring perceived support immediately following an interaction with a friend, there are some limitations to this measurement. First, participants completed the entire interaction before perceived support was measured, consistent with similar tasks (e.g., Priem et al., 2009). Pausing the interaction to complete these ratings before switching roles was deemed infeasible, as an evaluation of support midway through the interaction may have made issues of support more salient to participants, such that they modify their behaviour or attention during the latter half of the interaction. Accordingly, the participants’ ratings of support may reflect their experience during the entire interaction. Additionally, although a benefit of this work was measuring each participant’s perceptions of both the support they felt they received from the friend, and how supported they thought their friend felt, the extent to which we directly measured participants’ perceptions of support given is unclear. While we maintained a balance between the items measuring support received and support given, different results may have been obtained had we used items that more directly assessed perceptions of the support each participant gave.

While verbatim transcripts of the interactions provided a wealth of information regarding verbal responses, much information is conveyed through nonverbal channels that may have implications in the support process. Some types of information may be more clearly conveyed through verbal channels than others, which may have impacted our findings. For example, neutral or positive messaging may be enhanced by vocal tone and body language, which could modify the way these messages shape perceptions of support, while directly negative messaging may be more easily tracked through verbal channels only. Future work may seek to include measures of nonverbal communication in addition to verbal responses to create a more holistic understanding of the support process within young adult friendships.

#### Conclusion

This study provides new insights into the support process within young adult friendships, highlighting that both verbal responding and positive friendship quality are associated with perceptions of support within an interaction. The results indicate that actors’ overtly supportive verbal responses were associated with their perceptions of both giving and receiving greater support, but they were not associated with the friends’ perceptions of support. On the other hand, negative responding styles were linked to poorer perceptions of support by the friends, but not the participant themselves. Moreover, evaluating one’s friendship positively is associated with feeling more supported during those interactions. Importantly, while overtly positive and negative styles of verbal responding are still linked with support perceptions even when accounting for positive friendship quality, it is possible that friendship quality contributes to a greater proportion of perceptions of support than other specific verbal responses. In sum, it is perhaps noticeable when friends deliver poor support, while at the same time, the reservoir of friendship quality is a resource that promotes feeling supported by a friend.

## Supplemental material

Supplemental material - Say you’ll be there: Associations between observed verbal responses, friendship quality, and perceptions of support in young adult friendships Click here for additional data file. Supplemental material for Say you’ll be there: Associations between observed verbal responses, friendship quality, and perceptions of support in young adult friendships by Erin P. Macdonald, Thomas H. Khullar, Ella L. Vezina, Katya Santucci, John E. Lydon, Amanda J. Rose, and Melanie A. Dirks in Journal of Social and Personal Relationships.
