# Supplementary material for: Say you’ll be there: Associations between observed verbal responses, friendship quality, and perceptions of support in young adult friendships
Source: J Soc Pers Relat. 2023 Aug 17;40(12):4001–22. doi: 10.1177/02654075231195115 (PMC10695744; doi:10.1177/02654075231195115)
Supplement: Supplemental material - Say you’ll be there: Associations between observed verbal responses, friendship quality, and perceptions of support in young adult friendships [file sj-pdf-1-spr-10.1177_02654075231195115.pdf]

Examining Mean Differences of Speaking Order across Study Variables

In this study, pairs of friends completed the Problem Talk Task, where each friend took an 8-minute turn speaking about a current problem before switching. Speaking order (i.e., first or second) was randomly assigned. Actor Partner Interdependence Models (APIM) analyzed the associations between verbal responses from the listener, friendship quality, and perceptions of support following the interaction. Models specified indistinguishable dyads, given the same-gender friendships within dyads and random assignment of participant speaking order during the task. To ensure there were no distinguishable differences between speaking first or second, we completed 10 t-tests to assess mean differences across study variables (see Table S1). Across 10 t-tests examining mean differences, there was only one significant test; specifically, there was a mean difference in information/opinion responses between Participant 1 and Participant 2, $t(262) = 2.77$, $p = .006$. This test does not retain statistical significance after accounting for multiple testing using the Benjamini-Hochberg correction.

Supplemental Table S1

Results of T-Tests Examining Mean Differences on Study Variables as a Function of Speaking Order in Problem Talk Task.

Variable	*t*	*p*
Support Received	-0.15	.881
Support Given	0.95	.343
Friendship Quality	0.32	.750
Support Responses	0.72	.471
Acknowledgement + Prompting	0.43	.670
Information + Opinion	2.77	.006
Advice + Help	0.21	.787
Questions	-1.31	.192
Distracting Responses	-0.83	.408
Negative Engagement Responses	-0.16	.870

Note. Analyses were independent samples t-tests comparing participants who spoke first to those who spoke second. Degrees of freedom for all tests were 262.

Exploring Interactions between Verbal Response and Friendship Quality on Perceptions of Support

We conducted exploratory analyses to test whether the association between verbal responses and perceptions of support varied as a function of positive friendship quality. Interaction models utilized a linear mixed model within an APIM framework using the GAMLj package in Jamovi (Gallucci, 2019). To test the moderation within an APIM framework, we included both the actor and partner main effects. To reduce the number of interaction terms and improve model fit (e.g., Garcia et al., 2015), we included only interactions using the actor report of positive friendship quality, resulting in two possible interaction effects: verbal response (actor) *X* friendship quality (actor), and verbal response (partner) *X* friendship quality (actor). The results of all interaction models and simple slopes for significant interaction effects can be found in Tables S2 through S11.

Supplemental Table S2

Interaction between Negative Engagement Responses and Friendship Quality on Support Received and Support Given

	Support Received				Support Given			
	B	*SE*	*t*	*p*	*B*	*SE*	*t*	*p*
Intercept	5.80	0.06	91.11	< .001	5.15	0.06	80.47	< .001
Negative Engagement (*actor*)	-1.05	2.03	-0.52	.606	-3.27	2.01	-1.63	.105
Negative Engagement (*partner*)	-3.26	2.04	-1.60	.112	-4.41	2.02	-2.19	.030
Friendship Quality (*actor*)	0.64	0.10	6.11	< .001	0.58	0.10	5.67	< .001
Friendship Quality (*partner*)	0.14	0.11	1.28	.200	0.02	0.10	0.23	.821
Neg Eng actor*FQQ actor	8.39	3.44	2.44	.015	7.43	3.40	2.18	.030
Neg Eng partner*FQQ actor	-2.23	3.56	-0.63	.532	-2.55	3.51	-0.73	.468
Gender	0.11	0.13	0.84	.401	0.01	0.13	0.42	.967
First Language	-0.27	0.12	-2.29	.023	-0.19	0.12	-1.64	.102

Note. Gender coded 0 = male, 1 = female. First language coded 0 = language other than English, 1 = English.

Supplemental Table S3

Simple Slopes for Interaction Between Actor Effect of Negative Engagement and Friendship Quality

	Support Received				Support Given			
	B	*SE*	*t*	*p*	*B*	*SE*	*t*	*p*
FQQ Mean – 1SD	-6.07	3.42	-1.78	.077	-7.71	3.38	-2.28	.023
FQQ Mean	-1.05	2.04	-0.52	.606	-3.27	2.01	-1.63	.105
FQQ Mean + 1SD	3.97	2.28	1.74	.084	1.17	2.26	0.52	.603

Supplemental Table S4

Interaction between Information + Opinion Responses and Friendship Quality on Support Received and Support Given

	Support Received				Support Given			
	B	*SE*	*t*	*p*	*B*	*SE*	*t*	*p*
Intercept	5.81	0.06	89.83	< .001	5.16	0.06	79.77	< .001
Information + Opinion (*actor*)	-0.57	0.39	-1.44	.152	-0.35	0.39	-0.92	.361
Information + Opinion (*partner*)	0.30	0.39	0.78	.436	-0.81	0.38	-2.13	.034
Friendship Quality (*actor*)	0.65	0.10	6.25	< .001	0.61	0.10	6.08	< .001
Friendship Quality (*partner*)	0.10	0.10	0.98	.330	0.02	0.10	0.20	.841
Info actor*FQQ actor	0.46	0.72	0.63	.530	1.28	0.71	1.82	.071
Info partner*FQQ actor	-0.72	0.58	-1.24	.216	-1.20	0.56	-2.13	.034
Gender	0.12	0.13	0.96	.337	0.05	0.13	0.37	.712
First Language	-0.27	0.12	-2.31	.022	-0.19	0.12	-1.66	.098

Note. Gender coded 0 = male, 1 = female. First language coded 0 = language other than English, 1 = English.

Supplemental Table S5

Simple Slopes for Interaction Between Partner Effect of Information + Opinion Responses and Friendship Quality

	Support Received				Support Given			
	B	*SE*	*t*	*p*	*B*	*SE*	*t*	*p*
FQQ Mean – 1SD	0.73	0.51	1.43	.155	-0.09	0.50	-0.17	.862
FQQ Mean	0.30	0.39	0.78	.436	-0.81	0.38	-2.13	.034
FQQ Mean + 1SD	-0.13	0.53	-0.24	.812	-1.53	0.52	-2.95	.003

Supplemental Table S6

Interaction between Distracting Responses and Friendship Quality on Support Received and Support Given

	Support Received				Support Given			
	B	*SE*	*t*	*p*	*B*	*SE*	*t*	*p*
Intercept	5.81	0.06	90.88	< .001	5.15	0.06	80.14	< .001
Distract (*actor*)	0.71	0.45	1.58	.116	0.99	0.44	2.25	.025
Distract (*partner*)	-0.94	0.45	-2.07	.040	0.51	0.45	1.14	.255
Friendship Quality (*actor*)	0.62	0.10	6.03	< .001	0.57	0.10	5.63	< .001
Friendship Quality (*partner*)	0.13	0.10	1.22	.223	0.05	0.10	0.52	.606
Distract actor*FQQ actor	-0.93	.811	-1.14	.254	-1.86	0.80	-2.34	.020
Distract partner*FQQ actor	-0.09	0.60	-0.14	.886	0.31	0.59	0.53	.595
Gender	0.12	0.13	0.92	.358	0.06	0.13	0.43	.668
First Language	-0.28	0.12	-2.37	.018	-0.20	0.12	-1.74	.083

Note. Gender coded 0 = male, 1 = female. First language coded 0 = language other than English, 1 = English.

Supplemental Table S7

Simple Slopes for Interaction Between Actor Effect of Distracting Responses and Friendship Quality

	Support Received				Support Given			
	B	*SE*	*t*	*p*	*B*	*SE*	*t*	*p*
FQQ Mean – 1SD	1.26	0.66	1.92	.057	2.11	0.65	3.25	.001
FQQ Mean	0.71	0.45	1.58	.116	0.99	0.44	2.25	.025
FQQ Mean + 1SD	0.15	0.67	0.23	.821	-0.12	0.66	-0.19	.853

Supplemental Table S8

Interaction between Support Responses and Friendship Quality on Support Received and Support Given

	Support Received				Support Given			
	B	*SE*	*t*	*p*	*B*	*SE*	*t*	*p*
Intercept	5.81	0.06	94.79	< .001	5.17	0.06	80.85	< .001
Support Response (*actor*)	1.57	0.72	2.17	.031	1.34	0.72	1.86	.064
Support Response (*partner*)	1.25	.070	1.78	.077	0.83	0.70	1.19	.236
Friendship Quality (*actor*)	0.65	0.10	6.28	< .001	0.57	0.10	5.61	< .001
Friendship Quality (*partner*)	0.09	0.10	0.82	.413	0.01	0.10	0.06	.956
Support actor*FQQ actor	-1.68	1.38	-1.22	.223	0.88	1.41	0.63	.532
Support partner*FQQ actor	0.67	1.26	0.53	.596	-0.52	1.28	-0.40	.686
Gender	0.12	0.12	0.98	.331	0.03	0.13	0.25	.806
First Language	-0.29	0.12	-2.54	.012	-0.21	0.12	-1.80	.073

Note. Gender coded 0 = male, 1 = female. First language coded 0 = language other than English, 1 = English.

Supplemental Table S9

Interaction between Acknowledgement / Prompting Responses and Friendship Quality on Support Received and Support Given

	Support Received				Support Given			
	B	*SE*	*t*	*p*	*B*	*SE*	*t*	*p*
Intercept	5.82	0.06	91.37	< .001	5.17	0.07	79.01	< .001
Acknowledge / Prompt (*actor*)	-0.39	0.47	-0.84	.400	-0.30	0.46	-0.66	.510
Acknowledge / Prompt (*partner*)	-0.02	0.47	-0.04	.967	-0.11	0.46	-0.23	.820
Friendship Quality (*actor*)	0.61	0.10	5.90	< .001	0.55	0.10	5.41	< .001
Friendship Quality (*partner*)	0.09	0.10	0.89	.373	-0.01	0.10	-0.06	.952
Acknowledge actor*FQQ actor	1.24	0.70	1.78	.076	0.94	0.69	1.37	.171
Acknowledge partner*FQQ actor	-0.13	0.78	-0.17	.868	-0.18	0.77	-0.23	.817
Gender	0.16	0.13	1.25	.214	0.07	0.13	0.54	.590
First Language	-0.29	0.12	-2.46	.015	-0.21	0.12	-1.83	.069

Note. Gender coded 0 = male, 1 = female. First language coded 0 = language other than English, 1 = English.

Supplemental Table S10

Interaction between Advice / Help Responses and Friendship Quality on Support Received and Support Given

	Support Received				Support Given			
	B	*SE*	*t*	*p*	*B*	*SE*	*t*	*p*
Intercept	5.81	0.06	91.18	< .001	5.17	0.07	79.42	< .001
Advice / Help (*actor*)	0.06	0.67	0.09	.931	0.75	0.66	1.13	.259
Advice / Help (*partner*)	0.11	0.68	0.17	.868	-0.43	0.66	-0.65	.515
Friendship Quality (*actor*)	0.64	0.10	6.18	< .001	0.58	0.10	5.71	< .001
Friendship Quality (*partner*)	0.11	0.11	1.01	.314	0.01	0.10	0.11	.916
Advice / Help actor*FQQ actor	-0.71	1.16	-0.61	.543	-0.98	1.14	-0.86	.391
Advice / Help partner*FQQ actor	0.73	1.21	0.60	.549	1.81	1.19	1.52	.129
Gender	0.12	0.13	0.95	.345	0.04	0.13	0.30	.768
First Language	-0.30	0.12	-2.48	.014	-0.24	0.12	-1.99	.047

Note. Gender coded 0 = male, 1 = female. First language coded 0 = language other than English, 1 = English.

Supplemental Table S11

Interaction between Question Responses and Friendship Quality on Support Received and Support Given

	Support Received				Support Given			
	B	*SE*	*t*	*p*	*B*	*SE*	*t*	*p*
Intercept	5.81	0.06	91.25	< .001	5.17	0.06	80.56	< .001
Question (*actor*)	-0.79	0.62	-1.27	.205	-1.77	0.61	-2.91	.004
Question (*partner*)	0.06	0.62	0.09	.929	-0.46	0.61	-0.76	.451
Friendship Quality (*actor*)	0.62	0.10	5.99	< .001	0.54	0.10	5.38	< .001
Friendship Quality (*partner*)	0.10	0.10	0.99	.326	-0.02	0.10	-0.18	.860
Question actor*FQQ actor	0.61	0.90	0.68	.499	0.76	0.88	0.87	.388
Question partner*FQQ actor	-0.49	0.93	-0.53	.596	0.15	0.91	0.16	.872
Gender	0.14	0.13	1.06	.291	0.08	0.13	0.59	.556
First Language	-0.32	0.12	-2.67	.008	-0.26	0.12	-2.25	.025

Note. Gender coded 0 = male, 1 = female. First language coded 0 = language other than English, 1 = English.

Exploratory Models Assessing Individual Verbal Responses, Friendship Quality, and Perceptions of Support

To better understand the associations between individual verbal responses, positive friendship quality, and perceptions of support, we ran APIM models testing each of the seven verbal responses entered individually, along with positive friendship quality. Covariates included gender and English as a first language. The results of these models can be found in Tables S12 - S18.

Supplemental Table S12

Results of APIM of Support Responses and Friendship Quality

	Support Received				Support Given			
	B	*SE*	*t*	*p*	*B*	*SE*	*t*	*p*
Intercept	5.90	0.12	49.23	< .001	5.26	0.12	42.66	< .001
Support Responses (*actor*)	1.37	0.70	1.95	.052	1.47	0.69	2.12	.035
Support Responses (*partner*)	1.32	0.70	1.89	.060	0.77	0.69	1.11	.267
Friendship Quality (*actor*)	0.64	0.10	6.20	< .001	0.57	0.10	5.70	< .001
Friendship Quality (*partner*)	0.10	0.10	0.99	.322	-0.00	0.10	-0.00	.998
Gender	0.12	0.12	0.93	.351	0.04	0.13	0.28	.779
First Language	-0.28	0.11	-2.47	.014	-0.21	0.12	-1.85	.066

Note. Gender coded 0 = male, 1 = female. First language coded 0 = language other than English, 1 = English.

Supplemental Table S13

Results of APIM of Acknowledgement / Prompting Responses and Friendship Quality

	Support Received				Support Given			
	B	*SE*	*t*	*p*	*B*	*SE*	*t*	*p*
Intercept	5.89	0.12	47.65	< .001	5.25	0.13	41.70	< .001
Acknowledge + Prompt (*actor*)	-0.53	0.46	-1.16	.248	-0.41	0.45	-0.91	.365
Acknowledge + Prompt (*partner*)	0.03	0.46	0.06	.952	-0.06	0.45	-0.13	.894
Friendship Quality (*actor*)	0.62	0.10	6.00	< .001	0.56	0.10	5.50	< .001
Friendship Quality (*partner*)	0.10	0.10	0.96	.340	-0.00	0.10	-0.02	.987
Gender	0.14	0.13	1.07	.284	0.42	0.13	0.42	.672
First Language	-0.29	0.12	-2.48	.014	-0.22	0.12	-1.86	.063

Note. Gender coded 0 = male, 1 = female. First language coded 0 = language other than English, 1 = English.

Supplemental Table S14

Results of APIM of Information / Opinion Responses and Friendship Quality

	Support Received				Support Given			
	B	*SE*	*t*	*p*	*B*	*SE*	*t*	*p*
Intercept	5.89	0.12	47.56	< .001	5.24	0.12	42.04	< .001
Information + Opinion (*actor*)	-0.55	0.39	-1.40	.162	-0.27	0.38	-0.71	.479
Information + Opinion (*partner*)	0.33	0.39	0.85	.396	-0.75	0.38	-1.96	.051
Friendship Quality (*actor*)	0.65	0.10	6.24	< .001	0.59	0.10	5.84	< .001
Friendship Quality (*partner*)	0.11	0.10	1.02	.308	0.03	0.10	0.26	.792
Gender	0.13	0.13	1.00	.318	0.06	0.13	0.44	.661
First Language	-0.28	0.12	-2.39	.018	-0.21	0.12	-1.82	.069

Note. Gender coded 0 = male, 1 = female. First language coded 0 = language other than English, 1 = English.

Supplemental Table S15

Results of APIM of Advice / Help Responses and Friendship Quality

	Support Received				Support Given			
	B	*SE*	*t*	*p*	*B*	*SE*	*t*	*p*
Intercept	5.90	0.12	47.68	< .001	5.26	0.13	41.81	< .001
Advice + Help (*actor*)	-0.04	0.66	-0.06	.952	0.54	0.65	0.83	.407
Advice + Help (*partner*)	0.20	0.66	0.31	.760	-0.30	0.65	-0.45	.651
Friendship Quality (*actor*)	0.64	0.10	6.17	< .001	0.57	0.10	5.66	< .001
Friendship Quality (*partner*)	0.11	0.10	1.01	.310	0.01	0.10	0.07	.947
Gender	0.12	0.13	0.95	.341	0.04	0.13	0.31	.758
First Language	-0.29	0.12	-2.42	.016	-0.22	0.12	-1.84	.067

Note. Gender coded 0 = male, 1 = female. First language coded 0 = language other than English, 1 = English.

Supplemental Table S16

Results of APIM of Question Responses and Friendship Quality

	Support Received				Support Given			
	B	*SE*	*t*	*p*	*B*	*SE*	*t*	*p*
Intercept	5.90	0.12	47.84	< .001	5.26	0.12	42.71	< .001
Questions (*actor*)	-0.86	0.61	-1.39	.165	-1.82	0.60	-3.02	.003
Questions (*partner*)	0.15	0.61	0.24	.804	-0.44	0.60	-0.73	.464
Friendship Quality (*actor*)	0.63	0.10	6.09	< .001	0.55	0.10	5.46	< .001
Friendship Quality (*partner*)	0.10	0.10	0.96	.337	-0.02	0.10	-0.16	.873
Gender	0.13	0.13	1.05	.293	0.07	0.13	0.55	.580
First Language	-0.31	0.12	-2.63	.009	-0.26	0.12	-2.24	.026

Note. Gender coded 0 = male, 1 = female. First language coded 0 = language other than English, 1 = English.

Supplemental Table S17

Results of APIM of Distracting Responses and Friendship Quality

	Support Received				Support Given			
	B	*SE*	*t*	*p*	*B*	*SE*	*t*	*p*
Intercept	5.89	0.12	47.68	< .001	5.23	0.12	42.30	< .001
Distracting (*actor*)	0.71	0.45	1.60	.111	1.00	0.44	2.25	.025
Distracting (*partner*)	-0.89	0.45	-2.00	.046	0.54	0.44	1.22	.225
Friendship Quality (*actor*)	0.63	0.10	6.14	< .001	0.59	0.10	5.82	< .001
Friendship Quality (*partner*)	0.11	0.10	1.10	.273	0.02	0.10	0.24	.812
Gender	0.12	0.13	0.96	.340	0.07	0.13	0.52	.605
First Language	-0.28	0.12	-2.37	.018	-0.21	0.12	-1.80	.073

Note. Gender coded 0 = male, 1 = female. First language coded 0 = language other than English, 1 = English.

Supplemental Table S18

Results of APIM of Negative Engagement Responses and Friendship Quality

	Support Received				Support Given			
	B	*SE*	*t*	*p*	*B*	*SE*	*t*	*p*
Intercept	5.92	0.12	47.86	< .001	5.28	0.12	42.58	< .001
Negative Engagement (*actor*)	0.87	1.89	0.46	.646	-1.54	1.86	-0.83	.409
Negative Engagement (*partner*)	-3.59	1.89	-1.91	.058	-4.58	1.86	-2.46	.015
Friendship Quality (*actor*)	0.61	0.10	5.82	< .001	0.55	0.10	5.44	< .001
Friendship Quality (*partner*)	0.14	0.11	1.34	.183	0.03	0.10	0.30	.768
Gender	0.11	0.13	0.83	.406	*0.00*	0.13	0.01	.993
First Language	-0.30	0.12	-2.55	.011	-0.22	0.12	-1.89	.060

Note. Gender coded 0 = male, 1 = female. First language coded 0 = language other than English, 1 = English.

References

Garcia, R. L., Kenny, D.A., & Ledermann, T. (2015), Moderation in the actor–partner interdependence model. *Personal Relationships*, *22*(1), 8-29. https://doi.org/10.1111/pere.12060
